# My Early Years of Yeast Mitochondrial Genetics

**DOI:** 10.3390/microorganisms12102077

**Published:** 2024-10-17

**Authors:** Ian G. Macreadie

**Affiliations:** School of Science, RMIT University, Bundoora, VIC 3083, Australia; ian.macreadie@rmit.edu.au

**Keywords:** ATP synthase, mitochondrial DNA, radioactive labelling, petite mutants, *Saccharomyces cerevisiae*, selfish intron, yeast genetics, yeast molecular biology

## Abstract

There have been massive technological advances in molecular biology and genetics over the past five decades. I have personally experienced these advances and here I reflect on those origins, from my perspective, studying yeast mitochondrial genetics leading up to deciphering the functions of the mitochondrial genome. The yeast contributions commenced in the middle of the last century with pure genetics, correlating mutants with phenotypes, in order to discover genes, just like the early explorations to discover new lands. The quest was to explore the mitochondrial genome and find its genes and their products. It was most fortunate that DNA sequencing technologies became available in the late 1970s, and laboratories were restructured enormously to keep pace with the emerging technologies. There were considerable costs in equipping laboratories, purchasing ultracentrifuges and restriction endonucleases, and undertaking DNA sequencing; additionally, workers required special safety gear.

## 1. Introduction

**Figure 1:**
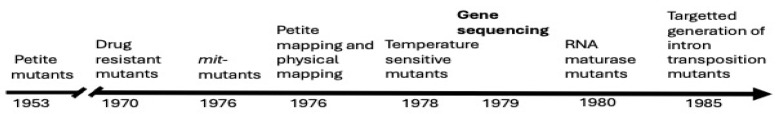
Timeline of important developments in yeast mitochondrial genetics.

The pace of technological advances over the past decades has been huge. I have written this reflective article to highlight the rapid changes that occurred in my specialty area, yeast mitochondrial genetics, from early advances on the genetics front to embracing the offerings that molecular biology brought about. My focus was on the mitochondrial genome of the simplest eukaryotic model organism, the budding yeast *Saccharomyces cerevisiae*. A brief timeline of the major advances is depicted in the timeline depicted in Figure 1.

My first experience with yeast research was in 1977 when I commenced work in Anthony W. Linnane’s group at Monash University in Clayton, Victoria, Australia. His group was a very large one in comparison to others in the world at that time. Under Linnane’s overall leadership, there were three sub-groups, led by senior staff H. Bruce Lukins, Philip Nagley and Sangkot Marzuki. Linnane’s efforts were focused on the biogenesis of mitochondria in yeast and many of his publications had “Biogenesis of mitochondria” in their title to indicate that they were part of a series. He had been involved in yeast mitochondrial studies since the 1960s and described the first mitochondrial drug-resistant mutants in 1970 [1].

In 1983, I had the opportunity to pursue a postdoc in another yeast mitochondrial genetics laboratory, that of Ronald Butow at the University of Texas Health Science Center at Dallas, Texas. At that stage, most of the functions of mitochondrial genes had been discovered; however, there were still some interesting problems to solve and I had the opportunity to participate further in yeast mitochondrial genetics.

## 2. Why Yeast?

Yeast is the simplest unicellular eukaryote and following its useful contributions to making beer, wine and bread, it also became useful in the study of genetics where mutants could be isolated, and phenotypes described. Useful laboratory strains became well developed for genetic studies and for deciphering biochemical pathways. The yeast used in these studies comprised haploid strains of both mating types (*MATa* and *MATα*) combined with mutations conferring auxotrophic nutritional requirements. Collections of strains were freely available throughout the world to the highly collegiate community of yeast researchers. Thus, genetics research could be readily performed on these strains.

**Figure 2:**
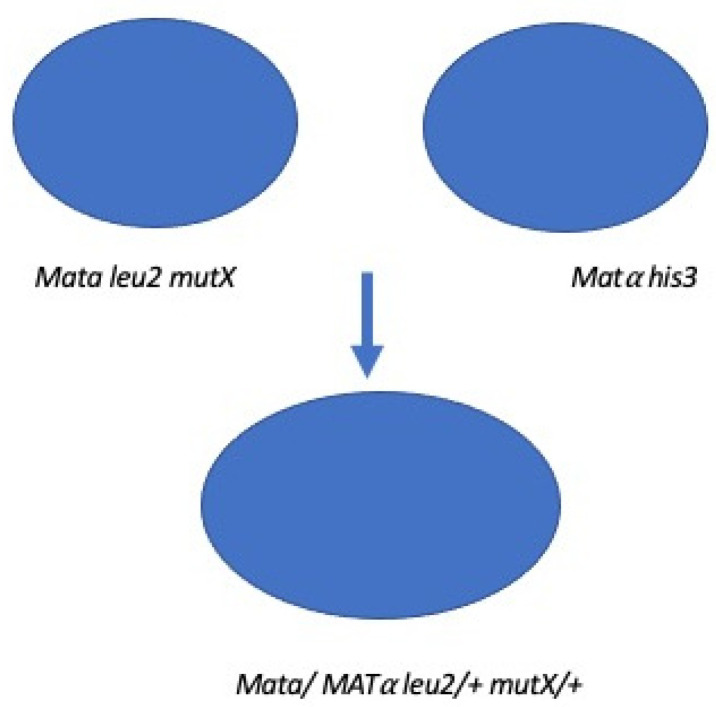
Schematic example of a genetic cross. Strains of opposite mating types are mixed in complete culture media. The first haploid strain requires leucine and the second requires histidine. After 5 h, the mixture is then placed onto minimal media (lacking leucine and histidine) for selection of diploids that have no requirement for histidine or leucine.

A simple example of a genetic cross is shown in Figure 2. In this hypothetical example, there is interest in knowing more about a new genetic marker shown as *mutX,* which confers resistance to drug X. Following the cross, diploid progeny are selected on minimal media. They are isolated as single colonies on minimal media in order to eliminate any possibility of the haploid strain being present. One can then examine the resistance to drug X in the diploid colonies. If all diploid colonies are sensitive to drug X, then *mutX* is a recessive mutation in a chromosomal gene. If all diploid strains are resistant to drug X, then *mutX* is a dominant mutation in a chromosomal gene.

Further genetic characterization can be performed by examining the haploid progeny after meiosis. Diploid yeast exhibits stable vegetative growth as diploids; however, they undergo meiosis when placed in a starvation medium. Starvation results in sporulation and meiotic cell divisions leading to the production of an ascus containing four spores. Using an enzyme to weaken the ascus, the four spores can be separated by a micro manipulator and the four spores are allowed to grow again. Testing the growth requirements of the four haploids will show 2:2 segregation of *leu2*, *his3* and *mutX* markers. However, after this process, there will be a recombination of the markers. If the markers are unlinked (as they would be if on different chromosomes), there will be a random reassortment of the markers. However, if *mutX* is near to *leu2* and on the same chromosome, there will be a high frequency of leucine auxotrophs that are resistant to drug X. The convenience of genetic markers in readily available strains, the culture of yeast on rich as well as synthetic defined culture media, and the ability to obtain and examine the products of meiosis, have made yeast an ideal tool for the establishment of robust genetics research around the globe.

## 3. Cytoplasmic Genes

The first pioneer of yeast mitochondrial genetics was the Moscow-born, Paris-based Boris Ephrussi, who described petite mutants in yeast, derived from a grande parent. In most strains of *Saccharomyces cerevisiae*, petites arose spontaneously at a frequency of up to 1%, although that frequency increased when yeast was grown in the presence of a mutagen such as ethidium bromide. Ephrussi [2] described that the petite mutation was due to a cytoplasmic factor. Later, Fred Sherman and Ephrussi [3] named this cytoplasmic factor the rho (ρ) factor. From a genetics perspective, grandes were ρ^+^. Examination of mitochondrial DNA (mtDNA) in two petite mutants showed that the bouyant density of their mtDNA was different from the wild-type strain [4], providing support for the ρ factor being the mitochondrial genome.

The petites resulted from a non-reverting deletion in their mitochondrial genome. Deletions occurred anywhere in the mitochondrial genome with the loss of multiple genes, resulting in all mitochondrial respiratory function being lost. This meant that unlike the parental strain, petites could no longer grow on a non-fermentable carbon source such as ethanol or glycerol. Therefore, petites were reduced in their growth on fermentable carbon sources and on solid media formed colonies that were of a small size.

The biochemical profile of one petite is the same as any other, so this limits their usefulness, except when it comes to genetic mapping. Petite mutants were useful in mitochondrial genetics since their genome could participate in genetic crosses to perform gene mapping. By assembling a library of petites retaining different portions of the mitochondrial genome, one could “rescue” mitochondrial mutants in a recombination process. I will come back to this.

## 4. The Function of the Mitochondrial Genome

Mitochondria have some special attributes. They are the powerhouse of the cell and are surrounded by a double membrane. The inner and outer membranes are sites of important biochemical functions like electron transport and oxidative phosphorylation. The proteins involved in these activities are multi-subunit complexes with most components being encoded by nuclear genes whose products are imported into the mitochondrion, but about one tenth of mitochondrial proteins are encoded and synthesised within mitochondria. Thus, the mitochondrion needs to have the capacity to replicate its genome, to transcribe it, and to translate it. How this happens was the reason for so much excitement in unravelling mitochondrial biogenesis by labs around the world.

**Figure 3:**
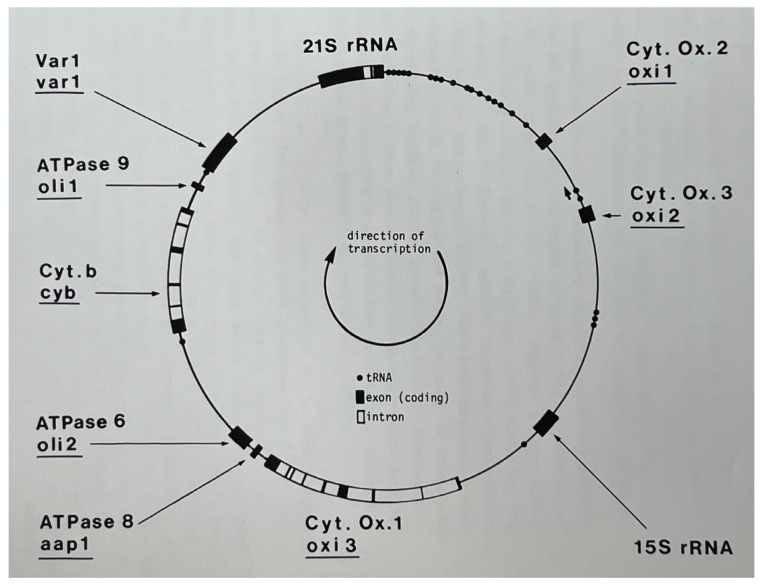
The yeast mitochondrial genome. Genes within the mitochondrial genome encode two rRNAs, one ribosomal protein Var1, tRNAs, three ATP synthase units, cytochrome *b*, and three subunits of cytochrome oxidase. In addition, there is a reading frame within the optional 21S rRNA intron that encodes an endonuclease (discussed in Part 8). From my PhD thesis [5].

We now know that the mitochondrial genome works in concert with the nuclear genome to produce multi-subunit complexes of the mitochondrion such as the ATP synthase, and cytochrome *b* and cytochrome oxidase, components of the electron transport chain. Without these complexes, there is no capacity for respiration. However, yeast exhibits another way of growth, fermentative growth. Thus, *S. cerevisiae* can grow in the absence of respiratory function. Figure 3 shows a map of the yeast mitochondrial genome.

There once was a question of whether mitochondria existed in the absence of respiratory function. The answer is that they do exist under all conditions, but they are not so morphologically distinct as when cells are growing through respiration.

## 5. Drug-Resistant, Temperature-Sensitive and *mit*- Mutants

The Monash lab had been involved in establishing the first mutations of mtDNA that conferred drug resistance [1]. For example, Linnane’s group found chloramphenicol, erythromycin and oligomycin resistances, which exhibited cytoplasmic genetics. In a cross between a drug-resistant and a drug-sensitive strain, the progeny were a mix of drug-resistant and drug-sensitive mutants, indicating cytoplasmic inheritance. For nuclear-encoded drug resistance, the four spores yielded after the meiotic divisions of a heterozygous diploid would exhibit 2:2 segregation. In contrast, for a cytoplasmic drug resistance mutation, all progeny would be the same after sporulation since the cytoplasmic factor, the mitochondria, go to all progeny.

The chloramphenicol and erythromycin resistances were associated with changes to rRNA genes, similar to what occurs in bacterial drug resistance to these antibiotics. This observation was used by some to support an idea that mitochondria were descended from bacteria. Other resistance mutations included oligomycin, antimycin, paromomycin, mucidin, mikamycin and venturicidin. These studies pre-dated today’s approach to genetic studies where genomes are known, and reverse genetics is used. They were pioneering since the early studies were performed with no sequence information.

When I arrived in Linnane’s group, I focussed on temperature-sensitive mutants and another class of mutants which totally eliminated the respiratory function, the *mit*- mutants. In contrast to petites, *mit*- mutants were large in size and their respiratory deficiency was due to a loss of just one mitochondrial function. The larger colony size of *mit*- mutants compared to petites may indicate that the *mit*- mutants have better energy conservation. Unlike petites, many *mit*- mutants resulted from point mutations and such mutants could revert to back to the wild type. In crosses of a *mit*- with a petite, if the petite contained the region of the DNA that was mutated in the *mit*- strain, genetic recombination could enable the recovery of a complete wild-type genome. Thus, respiratory function could be regained. This could readily be observed by crossing a *mit*- mutant with a library of petites and simply replica plating the mating mixture onto media with ethanol as the sole carbon source. Performing crosses of this type were known as petite mapping and it was a tremendous asset, helping in the rapid mapping of new mutations which were eagerly sought.

There were also mutations in the nuclear genome that could lead to the loss of mitochondrial function. These were known as *pet* mutants and they could be distinguished by genetic analysis.

The proteins of mitochondria were known through biochemical studies, and the products of the mitochondrial genome were known by a crafty approach of metabolic labelling. This involved growing the yeast strain in low-sulphate media and then adding radiolabelled sulphate while adding cycloheximide to inhibit all protein synthesis except for that going on in the mitochondrion. The mitochondrial proteins could then be displayed by autoradiography of an SDS-PAGE gel. The labelled products were few: three subunits of cytochrome oxidase, one subunit of cytochrome *b*, three subunits of the ATPase and one ribosomal protein called Var1.

The aim was to positively assign mutations to an effect in a mitochondrial protein, rRNA or tRNA, popularly known as gene function studies. Up to this point, all matches occurred in drug resistance genes but more discoveries were still to be made. One such discovery was mutations affecting ATPase subunit 8, found through the isolation of *mit*- mutations located between *oli2* and *oxi3*. To obtain mutants more easily without selection, one generally needs to increase mutation rates with the use of a selective mutagen. The mtDNA polymerase was made more error prone in the presence of manganese, so mtDNA mutagenesis was performed during fermentative growth (10% glucose without shaking) in the presence of MnCl_2_. The cells could then be spread onto solidified rich medium to isolate single colonies. I could then do a quick screen for colonies exhibiting respiratory deficiency, often by replica plating or by direct observation. In comparison to the parent strain, *mit*- colonies often had less colour, especially due to mitochondrial deficiencies such as reduced cytochrome levels. Also, the colonies were often sectored, because *mit*- mutants tended to have higher petite frequencies. Finally, they were tested for growth on YEPE (media comprising Yeast Extract, Peptone, Ethanol).

**Figure 4:**
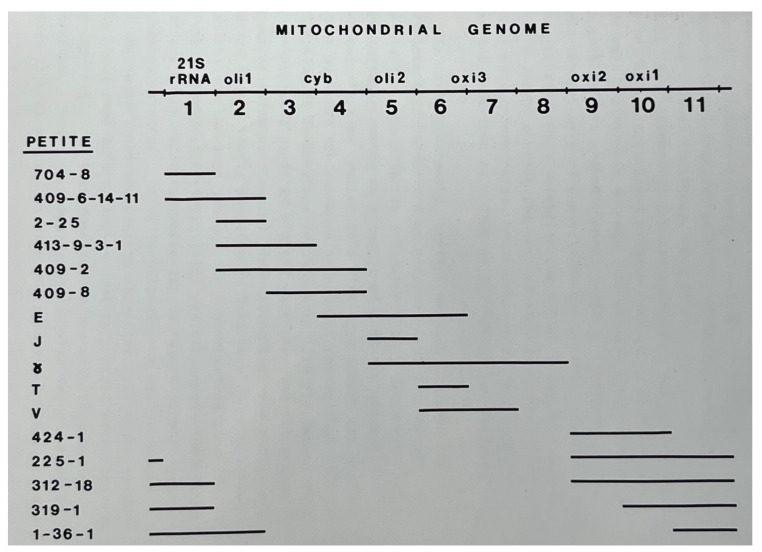
Schematic depiction of a library of petite mutants. In this diagram the mitochondrial genome is depicted, as it was known at the time, in a linear form and the portion of the mitochondrial genome retained by petites is also represented in a linear format. In petite mapping studies, if petites retained the region of the mitochondrial genome where a new mutation was found, the wild-type phenotype could be recovered. With this library of petites, the mutations in the mitochondrial genome could be mapped to the eleven portions of the mitochondrial genome. From my PhD thesis [5].

The respiratory deficient mutants were then crossed to a library of petites and respiratory function was restored if the petite mutant contained the mtDNA region that was mutated in the *mit*- mutant. Figure 4 depicts a petite library. In crosses between *mit*- mutants and petites, respiration could be recovered in progeny if the petite contained the region of the genome where the mutation was to be found.

## 6. A Transformation Through Molecular Biology Advances

In the mid-1970s, there started to be great advances in molecular analyses, in that we could start to look at the physical regions of DNA retained in petites. This involved isolating the total DNA and then separating mtDNA from nuclear DNA by overnight ultracentrifugation at 48,000 rpm. Addition of the DNA-binding chemical Hoescht 33258 was stronger to AT-rich DNA and aided the visualisation of the mtDNA band in the clear plastic centrifuge tube illuminated with long-wavelength UV light. In a darkened room, one could extract the mtDNA with a 22.5 gauge needle and syringe, passing the needle through the plastic centrifuge wall and into the band of mtDNA which could be drawn into the syringe. The lighter AT-rich mtDNA was above the thicker band of nuclear DNA.

Some of the earliest converging of the genetic and physical maps of the mitochondrial genome was performed by measuring the extent of DNA hybridization of genomes of petite mutants. An example of this is in the report by Linnane et al. [6]. However, this method was laborious, involved radiolabelling and did not reach the precision of that obtained with restriction fragment analysis, as described below.

A superior method emerged after restriction enzymes were discovered and utilised, although, because of their high costs in the mid-1970s, Phil Nagley’s group made some restriction endonucleases like *Eco*RI and *Pst*I. Purified mtDNA could be analysed by examining the sizes and positioning of restriction fragments sites it contained. This led to detailed maps of restriction enzyme sites in the mitochondrial genome being constructed. The size of the circular genome from respiratory-competent *S. cerevisiae* is 75 kb. This is surprisingly larger than the 16.6 kb mitochondrial genome which encodes thirteen proteins, two rRNAs and twenty-two tRNAs. The human mitochondrial genome is incredibly compact: some genes even lack a complete stop codon and rely on polyadenylation to add the missing nucleotides. However, the yeast mitochondrial genome contains AT-rich “spacer DNA” between genes, and curiously, there are introns in many mitochondrial genes.

The restriction sites in petite genomes helped to converge the genetic and physical maps [7].

## 7. A Transformation Through DNA Sequencing

Soon after that was the next big development: cloning DNA and DNA sequencing technologies. Maxam and Gilbert’s chemical cleavage method [8] was the method that I and others in Linnane’s lab used in the early 1980s. It required great skills in molecular techniques. The method required a ^32^P label to be placed at a single end of the DNA. One could start with a double-stranded DNA like a restriction fragment, label it at both ends with the Klenow fragment or a kinase. It was then either cut into two non-equal-sized fragments that could be separated on a gel and then extracted from the gel, or one could separate the two strands on a urea gel run at high temperature. Although of equal length, the strands could separate on the basis of slight differences in mass and perhaps secondary structure. The radiolabelled bands could be seen by exposure of the gels to an X-ray film. This was followed by cutting out radiolabelled bands, and electroelution of the DNA. The single end-labelled DNA could then be split up into four lots to perform the four limited chemical cleavages. The products were then run on a number of 80 cm long gels that were 0.3 mm thick so that DNA fragments in sizes ranging from 1 to ~1000 nucleotides in length could be separated. Samples were loaded onto the gels with the use of glass micropipettes that were siliconized and drawn out over the flame of a Bunsen burner so they were ultra-thin and could be used to load into the 0.3 mm gap. Samples going into gels were kept adjacent but separate with the use of a shark’s tooth spacer. Loading occurred through the use of one’s mouth pressure on the capillary, applied through a rubber tubing mouth pipette. I cringe thinking back on this in comparison to today’s laboratory health and safety standards. When working with ^32^P, we wore lead aprons, used PPE and worked behind Perspex shields. We had designated areas for radioactive work and these areas were monitored with Geiger counters. We also wore personal dosimeters on our chest and frequently wore finger dosimeters.

One glass plate was pre-treated to repel the gel while the other had an attractant. The gels were run at about 2000 volts and designed to run hot to denature the DNA so fragments would migrate according to their length. Following electrophoresis, the plates were carefully separated with the gel (hopefully) sticking to one glass plate. The gel was then induced to transfer to filter paper which was then dried under vacuum, and the dried gel was exposed to X-ray film in a light-tight cassette until an autoradiogram was developed. For sequencing with ^32^P, the exposure time to obtain results could take up to 2 weeks. If the result was not visible in 2 weeks (the half-life of ^32^P), the whole experiment would need to be repeated with a higher level of labelling. For this whole procedure to yield the desired sequence outcome, there needed to be sufficient radiolabelling. Losses of radiolabels were minimised by using siliconized tubes and pipettes. I remember once dropping a gel onto the floor. Although it broke into pieces, I went to great lengths to put it back together again so that my considerable efforts were not in vain. We also needed fresh supplies of ^32^P labelled nucleotide: supplies in lead containers and large eskies with dry ice usually arrived weekly from Amersham and DuPont.

The DNA sequence could be interpreted from the pattern of bands on the X-ray film, which was the size of a regular chest X-ray film. The film was developed in a hospital-type chest X-ray cassette that was developed with an enhancing screen at −70 °C. Most of our cassettes had had a former life in that role.

Some in the lab were starting on Sanger sequencing [9], but it required some knowledge of the sequence so that DNA primers could be designed. Sanger sequencing reconstructed DNA synthesis in vitro. Primers plus the Klenow fragment, dNTPs, a radiolabelled dATP, as well as dideoxy dNTPs to terminate DNA synthesis were employed. DNA was in four reactions with ddATP, ddCTP, ddTTP and ddGTP in each respective reaction. The reaction products were then analysed in the same way as those from chemical cleavage reactions. Sanger sequencing required far less skill in DNA preparation, but running gels still had the same level of difficulty. The use of ^35^S labelling meant that radioactivity hazards were not as high.

Further advances such as non-radioactive labelling became available much later. The use of four fluorescent labels coupled with capillary electrophoresis and sensitive fluorescence readers was a further huge transformation, although I did not get involved in that technology until the late 1980s. In more recent years, the outsourcing of these technologies has become routine and whole genome sequencing has become possible and inexpensive. What a transformation there has been in the recent decades.

It is also worth mentioning that petite mutants themselves were actually cloning vehicles: they amplified the mtDNA that they retained. On one occasion, I isolated the mtDNA from a petite that contained an ~1 kb region of the mitochondrial genome. That DNA was amplified in the petite and I used it for sequencing.

On another occasion I managed to use a restriction fragment from the mtDNA of an *aap1* mutant to determine the precise mutation in the mutant.

The genetics and molecular biology efforts led to a thorough analysis of the yeast mitochondrial genome, identifying tRNAs, the rRNAs, ATPase subunits 6, 8 and 9, cytochome b and three subunits of cytochrome oxidase. What was left to be done? The whole genome was sequenced!

## 8. Var1 and the Selfish Intron

In 1982, my contributions at Monash led to my being noticed at an international mitochondria conference held at Monash. I was invited by Ronald Butow to join his lab at the University of Texas Health Science Center at Dallas (also known as UT Southwestern Medical Center). This led to a productive time where I continued to be involved in yeast mitochondrial genome studies. One of the main tasks I faced was to understand ω, an optional mitochondrial intron whose sequence analysis revealed an open reading frame capable of encoding a protein of 235 amino acids [10]. ω was an optional region within the intron of the 21S rRNA gene. It conferred polarity (gene conversion) in crosses. While mitochondrial genes could exhibit normal genetic recombination, in a cross between an ω^+^ and an ω^−^ strain, almost all of the progeny became ω^+^. This had remained a mystery for a long time and was readily observed in strains that were marked with and without chloramphenicol and erythromycin resistance.

Introns occupied a considerable portion of the 75 kb genome of yeast mtDNA, but were totally absent in human mtDNA, which is very compact at just 17 kb: there are no spacers and no introns. Introns in yeast mtDNA were found in the genes encoding cytochrome *b* and cytochrome oxidase subunits and sometimes in the 21S rRNA gene. Interestingly, in strains having an intron in their 21S rRNA gene, there was an open reading frame (ORF).

There was the question as to whether this ORF was involved in the gene conversion. Mutants were needed to answer the question. Therefore, my quest was to obtain novel mutations in the open reading frame of the ω intron. I discussed how I might do this with Butow and colleagues, and he investigated options through his extensive network of colleagues working on yeast genetics. The yeast community has long been blessed by the collegiate nature of researchers in their extensive community. The strategy we devised was based on performing the manganese mutagenesis during yeast mating when replication of the ω intron was maximal. The strain to be used was a special strain capable of forming diploids that could be mated. To test whether gene conversion mutants had been made, the diploid was crossed once more to a chloramphenicol-resistant ω^−^ strain. The triploid progeny were then analysed for transmission of chloramphenicol sensitivity and the presence of the intron to identify strains that had lost the high frequency of intron transfer. This was analysed by a colony hybridisation screen with a radiolabelled intron probe.

**Figure 5:**
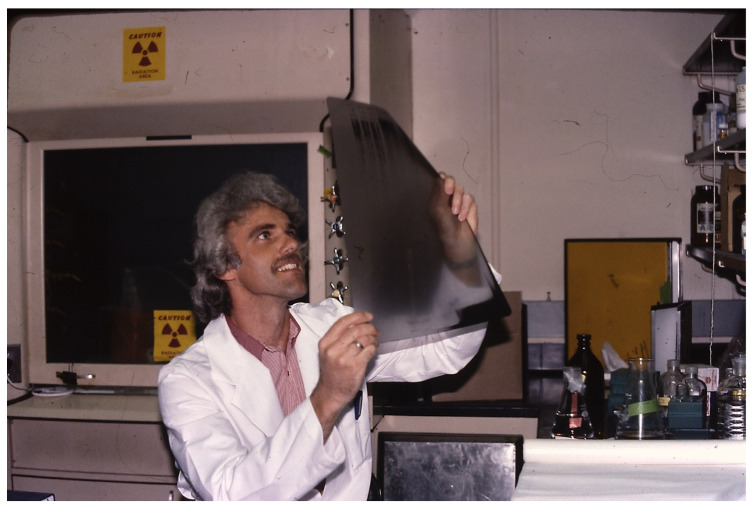
Me reading the sequence of the new mutations in the selfish intron.

Ultimately, around eight mutants defective in gene conversion were obtained and sequence analysis revealed they all had mutations in the intron’s open reading frame. My elation is shown in Figure 5.

In the meantime, Bernard Dujon (Centre de Génétique Moléculaire du CNRS Laboratoire associé à l’Université Pierre et Marie Curie, Gif Sur Yvette, France) was also at work on the ω^n^ strain and after much work found that a mutation in the open reading frame caused the loss of gene conversion. Our findings and Dujon’s findings were both published in 1985 in Cell [11,12], with Joe Sambrook writing a Nature commentary on the findings. The open reading frame had encoded an endonuclease, which Ron Butow named Scelase. The endonuclease recognised a sequence at the intron insertion site, cleaving it during crosses and allowing repair of the double-strand break from an ω^+^ template, accounting for the gene conversion. Colleaux et al. [13] produced the endonuclease and used it to define the 18 bp cleavage site, one of the most specific restriction sites known. While specific endonucleases were in high demand, the extremely rare recognition site of Scelase led to some referring to it as UScelase (useless).

Other work at UT Southwestern involved studies on the gene encoding the ribosomal protein Var1. Yeast mitochondrial genomes tend to be very AT-rich, which can aid their recovery after staining with Hoescht 33258 and buoyant density centrifugation. The Var1 gene was even more AT-rich, and in *Torulopsis glabrata* it was 90% AT, leading to thoughts that it might not be real. My task was to prove that the gene was indeed transcribed using a Northern blotting, the main technology of that time. It turned out to be very difficult to perform hybridisations with such high AT content and Butow kept suggesting that I did not want to make a career of repeating the experiment with so many failures. Eventually, I did resolve it, leading to a publication in J. Mol. Biol. [14]. At the same time, Peter Smooker had continued to work on a temperature-sensitive mutant of Var1 that I had characterised at Monash. Peter completed work on this mutant and this ended an era of work focused on defining the structure and function of mitochondrial genes [15].

## 9. Beyond Yeast Mitochondrial Gene Discovery

Once mitochondrial gene discovery was complete, efforts continued in yeast to manipulate the mitochondrial genome. Some turned to the nuclear genes that encoded mitochondrial proteins, while others set out to use the information provided by *Saccharomyces cerevisiae* to annotate mitochondrial genes in other organisms where equivalent genes could be readily analysed using bioinformatics tools.

As has happened time and time again, yeast research has underpinned great advances in our understanding of the ways cells function.

## Data Availability

No new data is presented.
